# Health Outcomes at School Age among Children Who Are HIV-Exposed but Uninfected with Detected Mitochondrial DNA Depletion at One Year

**DOI:** 10.3390/jcm9113680

**Published:** 2020-11-16

**Authors:** Audrey Monnin, Nicolas Nagot, Sabrina Eymard-Duvernay, Nicolas Meda, James K. Tumwine, Thorkild Tylleskär, Philippe Van de Perre, Jean-Pierre Molès

**Affiliations:** 1 Pathogenèse et Contrôle des Infections Chroniques, INSERM U1058, Université Montpellier, Etablissement Français du Sang, 34934 Montpellier, France; sabrina.eymard-duvernay@ird.fr (S.E.-D.); jean-pierre.moles@inserm.fr (J.-P.M.); 2 Pathogenèse et Contrôle des Infections Chroniques, INSERM U1058, Université Montpellier, Centre Hospitalier Universitaire, 34934 Montpellier, France; n-nagot@chu-montpellier.fr (N.N.); p-van_de_perre@chu-montpellier.fr (P.V.d.P.); 3 Centre MURAZ, Bobo-Dioulasso 01 01 B.P. 390, Burkina Faso; nicolas.meda@gmail.com; 4 Department of Paediatrics and Child Health, School of Medicine, College of Health Sciences, Makerere University, Kampala 7062, Uganda; kabaleimc@gmail.com; 5 Centre for International Health, Faculty of Medicine, University of Bergen, 5009 Bergen, Norway; thorkild.tylleskar@uib.no

**Keywords:** mitochondrial DNA, depletion, HIV-exposed uninfected children, lopinavir/ritonavir, lamivudine, growth, neurodevelopment, breastfeeding, Africa

## Abstract

Infant antiretroviral (ARV) prophylaxis given to children who are human immunodeficiency virus (HIV)-exposed but uninfected (CHEU) to prevent HIV transmission through breastfeeding previously proved its efficacy in the fight against the pediatric epidemic. However, few studies have investigated the short- and long-term safety of prophylactic regimens. We previously reported a decrease of mitochondrial DNA (mtDNA) content among CHEU who received one year of lamivudine (3TC) or lopinavir-boosted ritonavir (LPV/r) as infant prophylaxis. We aimed to describe mtDNA content at six years of age among these CHEU, including those for whom we identified mtDNA depletion at week 50 (decrease superior or equal to 50% from baseline), and to compare the two prophylactic drugs. We also addressed the association between mtDNA depletion at week 50 with growth, clinical, and neuropsychological outcomes at year 6. Quantitative PCR was used to measure mtDNA content in whole blood of CHEU seven days after birth, at week 50, and at year 6. Among CHEU with identified mtDNA depletion at week 50 (*n* = 17), only one had a persistent mtDNA content decrease at year 6. No difference between prophylactic drugs was observed. mtDNA depletion was not associated with growth, clinical, or neuropsychological outcomes at year 6. This study brought reassuring data concerning the safety of infant 3TC or LPV/r prophylaxis.

## 1. Introduction

In 2019, pediatric human immunodeficiency virus (HIV) infections accounted for about 9% of all new HIV infections worldwide, 90% of which were in sub-Saharan African countries [1]. According to the last 2020 report from the “Start free. Stay free. AIDS Free.” program, postnatal transmission of HIV accounted for 46% of new HIV infections in infants among 21 priority countries defined by the WHO in 2011 [2]. HIV transmission to infants during breastfeeding is mainly explained by the lack of adherence to the maternal antiretroviral (ARV) treatment or the absence of treatment [2,3]. Although considerable progress in accessing viral load and antiretroviral therapy has been made in many countries, with 85% of HIV pregnant women receiving ARV, efforts need to be reinforced in order to achieve the elimination of all postnatal transmission by 2030 [1].

All countries are now implementing a combination of universal maternal ARV treatments and children who are HIV-exposed but uninfected (CHEU) receive a prophylaxis using nevirapine (NVP) for 6 weeks in order to prevent mother-to-child transmission (MTCT) of HIV during breastfeeding [4]. Easier access to HIV viral load further improves this strategy by extending infant prophylaxis when breastfeeding mothers have an uncontrolled viral load. The type and duration of ARV prophylaxis given to breastfed CHEU was mostly empirical, monotherapy with NVP for 6 weeks within the current guidelines [4], dual therapy of zidovudine (AZT) plus NVP for 12 weeks at most for children who are at risk of acquiring HIV [4], or triple therapy (AZT/lamivudine (3TC)/NVP) as per Zambian guidelines for at least 12 weeks and to be stopped if the mother’s viral load is controlled, otherwise continued until a controlled viral load is reached, or four weeks after the cessation of breastfeeding [5].

Though the efficacy of infant prophylaxis has been demonstrated, few safety studies have been reported, especially considering that this additional ARV exposition applies to children who already show poorer health outcomes compared to children who are HIV-unexposed and uninfected (CHUU) [6]. A recent sub-analysis from the HIV Prevention Trials Network (HPTN) 046 trial study reported the safety profile of the extended prophylactic use of NVP from 6 weeks to 6 months on growth among breastfed CHEU followed until 18 months of age in comparison to CHEU receiving the current 6 weeks NVP recommendation [7]. Although standardized growth indicators (weight-for-age Z-score (WAZ), length-for-age Z-score, weight-for-length Z-score, and head circumference Z-score) declined over time in both groups of children compared to the general population, authors did not observe impaired growth in CHEU receiving the extended prophylaxis when compared to those receiving the standard NVP prophylaxis. CHEU receiving lopinavir/ritonavir (LPV/r) prophylaxis for one year in the PROMISE PEP trial exhibited stunted growth compared to those who received lamivudine (3TC) but normalized indicators at 6 years old [8]. They also showed an earlier asymptomatic adrenal dysfunction [9]. Furthermore, despite debates regarding neurological outcomes among CHEU, a recent study reported no neurodevelopmental differences between CHEU receiving NVP prophylaxis when compared to CHUU children in the first 60 months [10].

To date, there is no clear understanding of the underlying mechanisms that bring forward these key health differences, between CHEU and CHUU, and between CHEU receiving ARV prophylaxis or not. Previous works have reported differences in mitochondrial DNA (mtDNA) alterations such as a lower number of mtDNA copies per cell mainly resulting from the ARV exposure [11,12,13,14,15]. We recently reported altered mtDNA parameters among CHEU who received one year of 3TC or LPV/r monoprophylaxis [16]. More specifically, this acute toxicity is expressed by an mtDNA depletion (50% decrease of mtDNA content from baseline) at 6 months in comparison to CHEU who did not receive a prophylaxis. Whether this decrease of mtDNA content persists over time after discontinuation of ARV exposure remains less documented. Studies focusing on CHEU who did not receive ARV prophylaxis reported an increase or no change of mtDNA content at two years, but levels of mtDNA remained very low compared to that of HIV-unexposed children [11,15]. Only one U.S. cohort study was able to observe a recovery of mtDNA content at 5 years of age similar to control children [15]. The *Erythrocebus patas* monkey model is not reassuring regarding this point. Monkey offspring that were HIV-uninfected but exposed in utero to ARV showed progressive mtDNA depletion in the brain cortex from birth to one year (equivalent to 5 years for humans) for those exposed to AZT/3TC, AZT/didanosine (ddI), or d4T/3TC and compromised mitochondrial morphology at one and three years old (equivalent to 15 years old in humans) [17]. The latter finding raised concern for the neurodevelopment of CHEU treated or exposed to ARV who presented motor, cognitive, and language delays at one and two years of age as well as growth impairment in comparison with CHUU [18,19].

The objectives of our study were to describe at school age the mtDNA content from seven days of life to six years of age among CHEU who had received a one year LPV/r or 3TC prophylaxis, including those with previously reported mtDNA depletion at the end of the prophylaxis, and to compare the two drug regimens. We also addressed the association between mtDNA depletion at week 50 and growth, clinical, and neuropsychological outcomes at year 6.

## 2. Experimental Section

### 2.1. Study Design and Settings

The study is designed as an observational cross-sectional study, which recalled CHEU from Burkina Faso and Uganda between February 2017 and February 2018, who completed the final visit of the PROMISE PEP trial (NCT00640263) 5 years ago. Children, as well as their mothers or caregivers, were invited to the study site for a one or two day visit for a growth, clinical, and neuropsychological evaluation (PROMISE M&S trial, NCT03519503). Prior to enrollment, inclusion criteria were verified and the mother or legal guardian signed an informed consent form. The visit consisted of a consultation with a medical doctor and a psychologist [20].

### 2.2. Study Population

This study included CHEU who previously participated between November 2009 and May 2012 in the evaluation of the safety and the efficacy of LPV/r and 3TC pediatric formulations used as pre-exposure prophylaxis (PrEP) to prevent HIV acquisition during breastfeeding (PROMISE PEP trial) [21,22]. This includes CHEU from Burkina Faso, South Africa, Uganda, and Zambia who were randomly selected seven days after birth to receive either LPV/r or 3TC until one week after the cessation of breastfeeding, with a maximum duration of fifty weeks. Mothers were not eligible for an ARV treatment at the time of the trial (>350 CD4+ cells/mm^3^) but received ARV prophylaxis during pregnancy and labor as per national guidelines for the prevention of MTCT of HIV [23]. Children were eligible if they had available dried blood spots at day 7, week 50, and year 6. We randomly selected a total of 100 children from Burkina Faso and from Uganda with a 1:1 ratio of male and female children and a 1:1 ratio of 3TC and LPV/r prophylaxis in a two-step process; the first one selecting participants from the previous study evaluating the acute mitochondrial toxicity of the PrEP regimen [16] and a second one that completed the selection. Zambian and South African CHEU were not included in the present study because we had previously shown that mitochondrial parameters from whole blood interacted with platelet count and because the protocol did not include a blood sample repository, respectively.

### 2.3. Sample Collection and Processing

Capillary blood samples at day 7 and week 50 were collected by a heel prick directly on Whatman 903 cards. Venous blood collected in EDTA tubes were used for dried blood spot processing at year 6. All cards were stored with desiccant in a zipped pouch at −20 °C at the study sites. DNA extraction was performed from 3 mm diameter punches (*n* = 3) using the QIAamp DNA Blood Mini Kit (Qiagen, Hilden, Germany) following the manufacturers’ instructions. Extracted DNA was stored at −80 °C.

### 2.4. Neuropsychological Assessments

The Strengths and Difficulties Questionnaire (SDQ-25) was used to identify mental health symptoms and included fives scales of questions based on emotional problems, conduct problems, hyper activity, peer relationship problems, and prosocial behavior. The Test of Variable of Attention (TOVA) was used to measure attention and impulsivity in children. The Movement Assessment Battery for Children, second edition (MABC-2) was used to evaluate impairments in motor performance including manual dexterity, aiming and catching, and balance. The Kaufman Assessment Battery for Children, second edition (KABC-II) was used to evaluate cognitive abilities including sequential processing, simultaneous processing, and learning. Methodology for scores analysis and main findings are described elsewhere [20].

### 2.5. Mitochondrial DNA Copy Number per Cell Assay

mtDNA copy number per cell (MCN) was measured using two independent quantitative polymerase chain reactions using the QuickScanTM Mitox assay (Primagen, Amsterdam, The Netherlands) on a LightCycler^®^ 480 (Roche, Bâle, Switzerland) instrument [24], and was expressed as the ratio between mtDNA copy number of the *RNR2* gene and the copy number of the single-copy nuclear *SNRPA* gene. The sequences of the primers used were the following: for *RNR2* gene, forward 5′-GGGCTCTGCCATCTTAA-3′ and reverse 5′-GTAATCCAGGTCGGTTTCTA-3′; and for *SNRPA* gene, forward 5′-CGGCATGTGGTGCATAA-3′ and reverse 5′-TGCGCCTCTTTCTGGGTGTT-3′. The detailed protocol was previously described [16,24].

At each time point, we applied a stringent quality control of DNA (for details see [16]) by: (i) removing data with a cycle threshold (Ct) for *SNRPA* not comprised between the mean of all the Ct ± 2 standard deviations in order to filter for nuclear DNA degradation as previously reported [16,25], and (ii) by accounting for platelets and leucocytes counts because the MCN was quantified from whole blood. Platelet count correlation with MCN was assessed using Spearman’s rank order correlation test (if the absolute value of Rho was ≥0.3 with *p* ≤ 0.05, the site was excluded from the analysis). We also applied the formula of Hurtado allowing MCN adjustment on peripheral platelet and leucocyte counts [26]. If the difference between the raw and corrected data did not exceed 10%, the raw data were validated.

### 2.6. Statistical Analysis

Data are presented as means with their standard deviation (SD) or as medians with their interquartile range ([IQR]) for Gaussian or non-Gaussian continuous variables, respectively, and as frequencies with percentages for categorical variables.

In order to estimate a potential selection bias, we compared the characteristics of CHEU not including those enrolled in the study using chi-squared or Fisher’s exact tests as appropriate for categorical variables, and Student’s *t*-test or Wilcoxon Mann–Whitney test for continuous variables. Similarly, we compared the characteristics of CHEU with mtDNA depletion versus those without mtDNA depletion.

We compared MCN between the two prophylactic drugs and MCN between sites using the Wilcoxon Mann–Whitney test. Wilcoxon signed-rank test was used to compare MCN at day 7 and at week 50, and MCN at week 50 and at year 6. Differences in MCN between day 7 and week 50 and between week 50 and year 6 were compared between the two prophylactic drugs using Wilcoxon Mann–Whitney test. We used the chi-squared test to compare the proportion of CHEU with mtDNA depletion, defined as a 50% decrease of MCN at week 50 from its day 7 baseline value [16], between prophylactic drug regimens and between sites.

The association between mtDNA depletion at week 50 and growth indicators (WAZ, height-for-age Z-score (HAZ), and body mass index z-score (BMIZ)), neuropsychological test scores (SQD-25, TOVA, MABC-2, KABC-II), or hematological outcomes (platelet count) at year 6 was assessed using linear regression models. Global scores for three neuropsychological tests were used, and SDQ-25 questionnaire scores were square transformed to obtain a normal distribution. The association between mtDNA depletion at week 50 and “having a clinical or hospital consultation during the last years” or “having an abnormal lactate dehydrogenase (LDH) concentration at year 6” was assessed using log-binomial regressions. Analyses were adjusted for the gender of the child and the prophylactic drug.

Statistical analyses were performed using SAS studio (Copyright © 2012–2020 SAS Institute Inc., Cary, NC, USA). The forest plot was drawn using GraphPad software 7.0 (Copyright © 1992–2018).

### 2.7. Ethics Consideration

Written informed consents were obtained from the mother or the legal representative prior to enrollment in the PROMISE PEP trial (NCT00640263) and in PROMISE M&S trial (NCT03519503). The protocols were conducted in accordance with the Declaration of Helsinki and approved by the Ethical Committee for Health Research in Burkina Faso (#2008-039 and #2016-4-41) and the Uganda National Council for Science and Technology (#HS 470 and #HS1988).

## 3. Results

### 3.1. Study Population and Characteristics

The final study population after quality control assessment (see Result details in the Supplementary Materials) consisted of 86 CHEU with a median age of 6 years, equally distributed between both sites (Table 1). The male:female and prophylactic regimen (3TC:LPV/r) ratios were both approximately 1. The majority of children had normal growth indicators but severe-to-moderate stunting, wasting, and underweight were observed for 2 (2.4%), 4 (4.7%), and 3 (3.5%) children, respectively. The hematological parameters were normal for all children except 6 cases of anemia (7.0%) and 2 cases of neutropenia (2.3%). More than 97% of children had normal alanine aminotransferase (ALT) concentration, but LDH concentration was abnormal for 2/3 of children. Medical consultations since week 50 were also monitored. The characteristics of children enrolled in the study did not differ from those who were not enrolled except they had higher weights, heights, and SDQ-25 scores (Supplementary Table S1).

### 3.2. Overall mtDNA Content

At baseline, MCN was similar between treatment groups (*p* = 0.41) and between sites (*p* = 0.67), with a median MCN of 1082 and an interquartile range of [938;1253] (Table 2). At week 50, MCN showed a decreasing trend as compared to the matched baseline value (*p* = 0.23), with a median of 913 [655;1218] followed by an increase at year 6 (*p* = 0.10), ending up with a median of 955 [771;1192] at the end of the follow-up. No difference between treatment groups or between study sites was observed at week 50 (*p* = 0.42 and *p* = 0.86, respectively) and at year 6 (*p* = 0.37 and *p* = 0.29, respectively). The overall dynamic of mtDNA content is further described in Supplementary Table S2.

### 3.3. Mitochondrial DNA Depletion during the Follow-Up for CHEU

Because we previously reported a high frequency of children with mtDNA depletion at the end of the prophylaxis [16], we decided to focus on this specific population for the follow-up study. Characteristics of CHEU with or without mtDNA depletion were similar at year 6. Of note, more than 80% of CHEU with depletion presented an abnormal LDH concentration at year 6.

Among the 51 CHEU showing a decrease of MCN at week 50 out of the 86 CHEU analyzed (Supplementary Table S2), 17 cases (19.8%) of mtDNA depletion at W50 were reported, 11 in the 3TC arm (64.7%) and 6 in the LPV/r arm (35.3%) (*p* = 0.12) (Table 3). Cases of mtDNA depletion were nearly equally distributed among Burkina Faso and Uganda (*p* = 0.79). At year 6, all but one child with depletion at week 50 (94.1%) increased their MCN (*p* < 0.001; Table 3). Of these, 5 had an MCN above their baseline value with a median MCN of 1279 [926;1389] and 11 had an MCN below their baseline value with a median MCN of 808 [611;1294]. MCN increase from week 50 to year 6 was similar between treatment groups (*p* = 0.96). Of note, there was no difference between CHEU with mtDNA depletion between the PROMISE PEP and PROMISE M&S trials (Supplementary Table S3).

### 3.4. Growth, Clinical, and Neuropsychological Outcomes at Year 6 among CHEU with mtDNA Depletion

**Figure 1:**
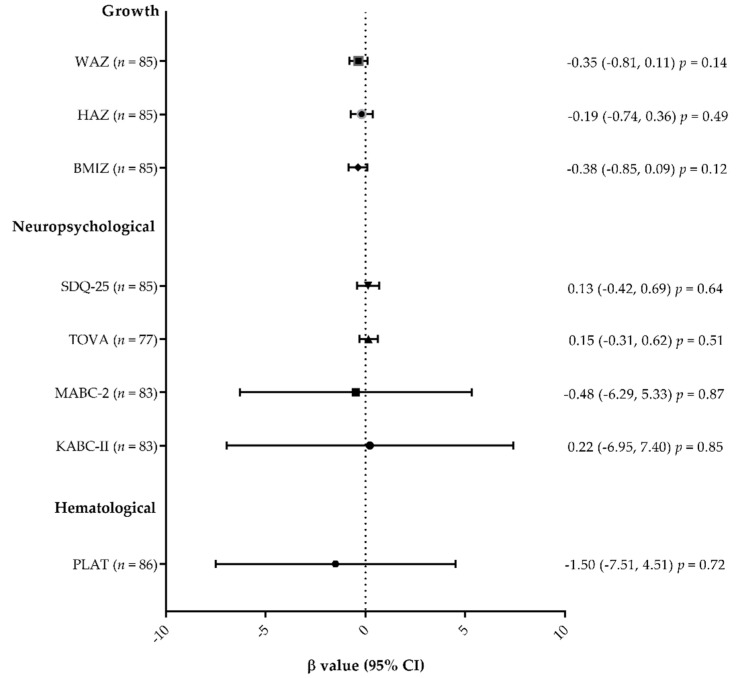
Forest plot of the association between anthropometric, hematological, and neuropsychological outcomes at year 6 with CHEU having a mtDNA depletion at week 50, using linear regressions. β values and confidence intervals are shown. Analyses were adjusted for gender and prophylactic treatment regimen. Abbreviations: WAZ, weight-for-age z-score; HAZ, height-for-age z-score; BMIZ, body mass index z-score; SDQ-25, Strengths and Difficulties Questionnaire; TOVA, Test of Variable of Attention; MABC-2, Movement Assessment Battery for Children—second edition; KABC-II, Kaufman Assessment Battery for Children—second edition; PLAT, platelet count; CI, confidence interval.

Linear regressions showed that mtDNA depletion at week 50 was not associated with poor growth outcomes (WAZ, HAZ, BMIZ), nor was it associated with lower scores on the neuropsychological tests or the platelet count at year 6 (Figure 1). Log-binomial regressions showed that mtDNA depletion was also not associated with medical consultations that had occurred since week 50 or with abnormal LDH concentration at year 6 (Table 4).

## 4. Discussion

Safety is the utmost priority for prevention interventions targeting a large number of persons. Supplementary ARV exposure early in life during prevention of MTCT HIV programs may increase the risk of mtDNA genotoxicity. Overall, the decrease of MCN previously observed during the first year of life among CHEU who received 3TC or LPV/r to prevent postnatal transmission of HIV through breastfeeding was not persistent five years after prophylaxis discontinuation. Furthermore, we demonstrated that mtDNA depletion at week 50 in children had no long-term impact on growth, clinical, and neuropsychological outcomes at year 6. Overall, these findings are very reassuring for different child populations: (i) most of the living CHEU (more than 15 million) who had been exposed in utero to 3TC, (ii) to the new CHEU who are an increasing population (about 1 million a year), and (iii) to HIV-infected children who initiated LPV/r- and/or 3TC-based treatment early in life.

We previously described mtDNA depletion at week 50 among CHEU receiving LPV/r or 3TC prophylaxis and identified male gender as a possible risk factor for being mtDNA depleted at one year of age [16]. The proportion of CHEU with mtDNA depletion was not different between the two random selections when restraining only to Burkina Faso and Uganda participants (28/91 (30.8% (95% CI: 22.2–40.9)) for the previous study versus 17/86 (19.8% (95% CI: 12.3–29.4)) for the current study; *p* = 0.09, chi-squared test). Although the prevalence was lower, confidence intervals overlapped. The proportion of CHEU with depletion in the LPV/r arm and in the 3TC arm also did not differ between the two studies (*p* = 0.10 and *p* = 0.46, respectively, chi-squared test). Gender was equally distributed among CHEU with mtDNA depletion between the two studies (33 males versus 25 females for the previous study and 8 males versus 9 females for the current study; *p* = 0.47, chi-squared test).

Upon drug discontinuation, we observed an increasing trend in MCN in the whole population between one and 6 years of age. Increase of mtDNA content was previously observed in a U.S. cohort of CHEU exposed in utero to AZT or AZT/3TC [15]. Authors reported a rate of increase of 60 copies per cell per year. Herein, this rate was lower with an overall increase of 8.4 copies per cell per year (11.8 in the 3TC arm). However, in the U.S. study, ARV exposure was shorter since children were not breastfed and did not receive ARV prophylaxis.

The association of these health outcomes and a possible link to mitochondrial dysfunction is currently not established. A decrease of mtDNA content per cell leads to a low production of cell energy, which impacts organs requiring high energetic supply such as muscles, the heart, and the brain, and all of the others to a lower extent. In 2003, Barret et al. described neurologic symptoms including motor abnormalities and lower cognitive performance not only among CHEU with established mitochondrial dysfunction but also among those with a suspicion of mitochondrial impairment based on observation of persistent hyperlactatemia and impaired mitochondrial respiratory-chain enzymatic activity [27]. Another study, although not showing an association, identified CHEU with possible mitochondrial dysfunction based on neurologic manifestations including febrile or afebrile seizures and delay in cognitive development [28]. To our knowledge, only one study reported lower height-for-age Z-scores among HIV-infected children with lower mtDNA content and decreased complex IV enzymatic activity from the mitochondrial respiratory chain [29]. Children herein who accumulated ARV exposure during pregnancy and during the first year of life, had few health problems, growth impairment, or hematological abnormalities at six years of age. Furthermore, those who experienced mtDNA depletion at one year had the same growth, clinical, and neuropsychological outcomes at age 6 as the others. However, serum lactate concentration was abnormally elevated for two thirds of children, including 14/17 (82.3%) of those with mtDNA depletion. Hyperlactatemia is commonly used as a surrogate marker of mitochondrial dysfunction. Elevated serum lactate was observed in CHEU during the first year of life but association with MCN has not been reported [27,30,31,32,33]. In line with this, neither mtDNA depletion at week 50 nor MCN at year 6 were associated with hyperlactatemia in this study.

This study is unique because it describes (1) the longitudinal mtDNA content among CHEU older than all previous studies investigating MCN, which stop at school age, and (2) the evaluation of health outcomes at distance of PrEP regimen among African children, which represented 90% of the CHEU worldwide [1]. Furthermore, this study benefited from the randomization of the initial clinical trial. Several limitations to our study can be mentioned. First, CHEU from two sites of the initial trial could not be analyzed. In fact, a correlation between platelet count and MCN was previously observed during the first year of life for children from Zambia, which biased the analysis [16]. Follow-up of South African children was also not possible because samples at year 6 were not banked. Secondly, we did not have intermediate points between week 50 and year 6 that would have made the assessment more accurate. Thirdly, medical events, such as a history of clinical consultations or hospitalizations, were self-declared and not certified by a medical record. Finally, these children had a unique exposure to HIV and ARV, which makes the findings difficult to apply to other child populations. However, these findings can help to discriminate the true drug side effect from the HIV infection.

## 5. Conclusions

Altogether, adding ARV prophylaxis during the at-risk period of HIV transmission to CHEU, had no long-term health consequences for those who had mtDNA depletion at the end of ARV exposure. However, the current strategies for the prevention of MTCT use different drugs and treatment regimens, either for the mother or the child, suggesting that such mtDNA assessment and follow-up at distance of PrEP period is worth replicating.

## Figures and Tables

**Table 1:** Characteristics of children who are human immunodeficiency virus (HIV)-exposed but uninfected (CHEU) enrolled in the study.

Characteristic	CHEU Enrolled (*n* = 86)
Sociodemographics	
Age (in years); median [IQR]	6.0 [5.0–6.0]
Site; *n* (%)	
Burkina Faso	43 (50.0)
Uganda	43 (50.0)
Gender; *n* (%)	
Male	44 (51.2)
Anthropometrics; mean ± SD	
Weight (kg)	19.9 ± 2.9 ^†^
Height (cm)	115.7 ± 6.2 ^†^
WAZ	−0.5 ± 0.9 ^†^
HAZ	−0.3 ± 1.0 ^†^
BMIZ	−0.4 ± 0.9 ^†^
Hematology	
Hemoglobin concentration (g/dL); *n* (%)	
Normal > 10.4	80 (93.0)
Anemia ≤ 10.4	6 (7.0)
Mild [10.4–9.5]	5 (5.8)
Moderate [9.5–8.5]	1 (1.2)
Platelet count (10^3^/mm^3^); mean ± SD	358.4 ± 110.2
Platelet count (10^3^/mm^3^); *n* (%)	
Normal > 125	86 (100.0)
Leucocyte count (10^3^/mm^3^); *n* (%)	
Normal > 2.5	86 (100.0)
Neutrophil count (10^3^/mm^3^); *n* (%)	
Normal > 1.0	84 (97.7)
Neutropenia ≤ 1.0	2 (2.3)
Mild [1.0–0.79]	2 (2.3)
Biochemistry; *n* (%)	
LDH concentration (×ULN)	
Normal < ULN	27 (31.4)
Abnormal ≥ ULN	59 (68.6)
Mild [ULN-2 × ULN]	59 (68.6)
ALT concentration (U/L)	
Normal < 1.25 × ULN	84 (97.7)
Abnormal ≥ 1.25 × ULN	2 (2.3)
Mild [1.25–2.5] × ULN	1 (1.16)
Moderate [2.5–5.0] × ULN	1 (1.16)
Medical events; *n* (%)	
Clinical consultation without admission during the last year	
Yes	56 (65.9) ^†^
Hospital admission since week 50	
Yes	26 (31.3) ^§^
Child ARV prophylaxis	
Lamivudine	41 (47.7)
Lopinavir/ritonavir	45 (52.3)
Neuropsychological assessment	
SDQ-25; median [IQR]	6.0 [3.0;9.0] ^†^
TOVA; mean ± SD	2.2 ± 0.8 ^£^
MABC-2; mean ± SD	78.2 ± 10.7 ^§^
KABC-II; mean ± SD	48.9 ± 12.9 ^§^

^†^ One missing value, ^£^ nine missing values, ^§^ three missing values. Abbreviations: CHEU, children who are HIV-exposed but uninfected; IQR, interquartile range; SD, standard deviation; WAZ, weight-for-age z-score; HAZ, height-for-age z-score; BMIZ, body mass index z-score; LDH, lactate dehydrogenase; ALT, alanine aminotransferase; ULN, under limit of normal; ARV, antiretroviral; SDQ-25, Strengths and Difficulties Questionnaire; TOVA, Test of Variable of Attention; MABC-2, Movement Assessment Battery for Children—second edition; KABC-II, Kaufman Assessment Battery for Children—second edition.

**Table 2:** Mitochondrial DNA content at baseline, week 50, and year 6.

			Burkina Faso		Uganda		All
Follow-Up Time Point	PrEP	*n*	Median MCN [IQR]	*n*	Median MCN [IQR]	*n*	Median MCN [IQR]
day 7	3TC	20	1014 [856;1415]	21	1083 [950;1145]	41	1083 [950;1145]
	LPV/r	23	1061 [938;1436]	22	1084 [992;1198]	45	1084 [992;1198]
	All	43	1053 [898;1436]	43	1083 [960;1180]	86	1082 [938;1253]
week 50	3TC	20	861 [658;1183]	21	888 [470;1209]	41	868 [603;1192]
	LPV/r	23	908 [710;1300]	22	993 [742;1319]	45	972 [742;1300]
	All	43	876 [660;1192]	43	972 [563;1261]	86	913 [655;1218]
year 6	3TC	20	847 [620;1079]	21	1124 [836;1183]	41	926 [717;1157]
	LPV/r	23	997 [720;1389]	22	957 [812;1182]	45	960 [801;1279]
	All	43	904 [689;1157]	43	976 [812;1183]	86	955 [771;1192]

Abbreviations: PrEP, pre-exposure prophylaxis; MCN, mitochondrial DNA copy number per cell; IQR, interquartile range; 3TC, lamivudine; LPV/r, lopinavir/ritonavir.

**Table 3:** Mitochondrial DNA depletion among CHEU by time point.

			Burkina Faso	Uganda	All
Follow-Up Time Point	Group	PrEP	*n* (%)	*n* (%)	*n* (%)
week 50	Depletion of mtDNA at week 50	3TC	5 (55.6)	6 (75.0)	11 (64.7)
		LPV/r	4 (44.4)	2 (25.0)	6 (35.3)
		All	9 (52.9)	8 (47.1)	17 (100.0)
year 6	Increase of MCN at year 6 from week 50	3TC	4 (50.0)	6 (75.0)	10 (62.5)
		LPV/r	4 (50.0)	2 (25.0)	6 (37.5)
		All	8 (50.0)	8 (50.0)	16 (100.0)
	Decrease of MCN at year 6 from week 50	3TC	1 (100.0)	0	1 (100.0)
		LPV/r	0	0	0
		All	1 (100.0)	0	1 (100.0)

Abbreviations: mtDNA, mitochondrial DNA; PrEP, pre-exposure prophylaxis; 3TC, lamivudine; LPV/r, lopinavir/ritonavir.

**Table 4:** Log-binomial regressions for the association between medical and biochemical outcomes at year 6 with CHEU having a mtDNA depletion at week 50.

**Clinical Consultation without Admission during the Last Year**				
	***n***	**Adjusted PR**	**95% CI**	***p* Value**
mtDNA depletion at week 50				
Yes	17	0.76	0.50–1.15	0.19
**Hospital Admission Since the PROMISE-PEP Trial**				
	***n***	**Adjusted PR**	**95% CI**	***p* Value**
mtDNA depletion at week 50				
Yes	17	0.69	0.27–1.73	0.43
**Abnormal LDH Concentration at Y6**				
	***n***	**Adjusted PR**	**95% CI**	***p* Value**
mtDNA depletion at week 50				
Yes	17	1.20	0.89–1.60	0.23

Abbreviations: PR, prevalence ratio; CI, confidence interval; mtDNA, mitochondrial DNA; LDH, lactate dehydrogenase; Y6, year 6.

## Supplementary Materials

The following are available online at https://www.mdpi.com/2077-0383/9/11/3680/s1, Result details: Quality control of DNA, Table S1: Characteristics of CHEU from the PROMISE PEP trial enrolled in the analysis and CHEU not enrolled, Table S2: Overall dynamic of mtDNA content, Table S3: Characteristics at W50 of the CHEU with depletion between the PROMISE PEP and the PROMISE M&S trials.

## References

[1] UNAIDS. http://aidsinfo.unaids.org/.

[2] UNAIDS Progress towards the Start Free, Stay Free, AIDS Free Targets 2020 Report. https://www.unaids.org/sites/default/files/media_asset/start-free-stay-free-aids-free-2020-progress-report_en.pdf.

[3] Millar J.R., Bengu N., Fillis R., Sprenger K., Ntlantsana V., Vieira V.A., Khambati N., Archary M., Muenchhoff M., Groll A. (2020). High-frequency failure of combination antiretroviral therapy in paediatric HIV infection is associated with unmet maternal needs causing maternal non-adherence. EClinicalMedicine.

[4] World Health Organization (2016). Consolidated Guidelines on the Use of Antiretroviral Drugs for Treating and Preventing HIV Infection: Recommendations for a Public Health Approach.

[5] Ministry of Health Zambia Consolidated Guidelines for Treatment and Prevention of HIV Infection. https://www.moh.gov.zm/wp-content/uploads/filebase/Zambia-Consolidated-Guidelines-for-Treatment-and-Prevention-of-HIV-Infection-2020.pdf.

[6] Desmonde S., Goetghebuer T., Thorne C., Leroy V. (2016). Health and survival of HIV perinatally exposed but uninfected children born to HIV-infected mothers. Curr. Opin. HIV AIDS.

[7] Onyango-Makumbi C., Owora A.H., Mwiru R.S., Mwatha A., Young A.M., Moodley D., Coovadia H.M., Stranix-Chibanda L., Manji K., Maldonado Y. (2019). extended prophylaxis with nevirapine does not affect growth in hiv-exposed infants. J. Acquir. Immune Defic. Syndr..

[8] Blanche S., Tylleskär T., Peries M., Kankasa C., Engebretsen I., Meda N., Tumwine J.K., Singata-Madliki M., Mwiya M., Van de Perre P. (2019). Growth in HIV-1-exposed but uninfected infants treated with lopinavir-ritonavir versus lamivudine: A secondary analysis of the ANRS 12174 trial. Lancet HIV.

[9] Kariyawasam D., Peries M., Foissac F., Eymard-Duvernay S., Tylleskär T., Singata-Madliki M., Kankasa C., Meda N., Tumwine J.K., Mwiya M. (2019). Lopinavir-Ritonavir Impairs Adrenal Function in Infants. Clin. Infect. Dis..

[10] Boivin M.J., Maliwichi-Senganimalunje L., Ogwang L.W., Kawalazira R., Sikorskii A., Familiar-Lopez I., Kuteesa A., Nyakato M., Mutebe A., Namukooli J. (2019). Neurodevelopmental effects of ante-partum and post-partum antiretroviral exposure in HIV-exposed and uninfected children versus HIV-unexposed and uninfected children in Uganda and Malawi: A prospective cohort study. Lancet HIV.

[11] Poirier M.C., Divi R.L., Al-Harthi L., Olivero O.A., Nguyen V., Walker B., Landay A.L., Walker V.E., Charurat M., Women and Infants Transmission Study (WITS) Group (2003). Long-term mitochondrial toxicity in HIV-uninfected infants born to HIV-infected mothers. J. Acquir. Immune Defic. Syndr..

[12] Divi R.L., Walker V.E., Wade N.A., Nagashima K., Seilkop S.K., Adams M.E., Nesel C.J., O’Neill J.P., Abrams E.J., Poirier M.C. (2004). Mitochondrial damage and DNA depletion in cord blood and umbilical cord from infants exposed in utero to Combivir. AIDS.

[13] Divi R.L., Leonard S.L., Kuo M.M., Nagashima K., Thamire C., St. Claire M.C., Wade N.A., Walker V.E., Poirier M.C. (2007). Transplacentally exposed human and monkey newborn infants show similar evidence of nucleoside reverse transcriptase inhibitor-induced mitochondrial toxicity. Environ. Mol. Mutagen..

[14] Hernàndez S., Morén C., López M., Coll O., Cardellach F., Gratacós E., Miró O., Garrabou G. (2012). Perinatal outcomes, mitochondrial toxicity and apoptosis in HIV-treated pregnant women and in-utero-exposed newborn. AIDS.

[15] Aldrovandi G.M., Chu C., Shearer W.T., Li D., Walter J., Thompson B., McIntosh K., Foca M., Meyer W.A., Ha B.F. (2009). Antiretroviral exposure and lymphocyte mtDNA content among uninfected infants of HIV-1-infected women. Pediatrics.

[16] Monnin A., Nagot N., Peries M., Vallo R., Meda N., Singata-Madliki M., Tumwine J.K., Kankasa C., Ngandu N., Goga A. (2020). Mitochondrial DNA parameters in blood of infants receiving lopinavir/ritonavir or lamivudine prophylaxis to prevent breastfeeding tansmission of HIV-1. J. Clin. Med..

[17] Liu Y., Park E.S., Gibbons A.T., Shide E.D., Divi R.L., Woodward R.A., Poirier M.C. (2016). Mitochondrial compromise in 3-year old patas monkeys exposed in utero to human-equivalent antiretroviral therapies. Environ. Mol. Mutagen..

[18] McHenry M.S., McAteer C.I., Oyungu E., McDonald B.C., Bosma C.B., Mpofu P.B., Deathe A.R., Vreeman R.C. (2018). Neurodevelopment in Young Children Born to HIV-Infected Mothers: A Meta-analysis. Pediatrics.

[19] Wedderburn C.J., Evans C., Yeung S., Gibb D.M., Donald K.A., Prendergast A.J. (2019). Growth and Neurodevelopment of HIV-Exposed Uninfected Children: A Conceptual Framework. Curr. HIV/AIDS Rep..

[20] United States National Library of Medicine. https://clinicaltrials.gov/ct2/show/NCT03519503?term=promise+m%26s&draw=2&rank=1.

[21] Nagot N., Kankasa C., Meda N., Hofmeyr J., Nikodem C., Tumwine J.K., Karamagi C., Sommerfelt H., Neveu D., PROMISE-PEP group (2012). Lopinavir/Ritonavir versus Lamivudine peri-exposure prophylaxis to prevent HIV-1 transmission by breastfeeding: The PROMISE-PEP trial Protocol ANRS 12174. BMC Infect. Dis..

[22] Nagot N., Kankasa C., Tumwine J.K., Meda N., Hofmeyr G.J., Vallo R., Mwiya M., Kwagala M., Traore H., Sunday A. (2016). Extended pre-exposure prophylaxis with lopinavir-ritonavir versus lamivudine to prevent HIV-1 transmission through breastfeeding up to 50 weeks in infants in Africa (ANRS 12174): A randomized controlled trial. Lancet.

[23] World Health Organization (2006). Antiretroviral Drugs for Treating Pregnant Women and Preventing HIV Infection in Infant: Towards Universal Access: Recommendations for a Public Health Approach.

[24] Timmermans E.C., Tebas P., Ruiter J.P.N., Wanders R.J.A., de Ronde A., de Baar M.P. (2006). Real-time nucleic acid sequence-based amplification assay to quantify changes in mitochondrial DNA concentrations in cell cultures and blood cells from HIV-infected patients receiving antiviral therapy. Clin. Chem..

[25] Ashar F.N., Moes A., Moore A.Z., Grove M.L., Chaves P.H.M., Coresh J., Newman A.B., Matteini A.M., Bandeen-Roche K., Boerwinkle E. (2015). Association of mitochondrial DNA levels with frailty and all-cause mortality. J. Mol. Med..

[26] Hurtado-Roca Y., Ledesma M., Gonzalez-Lazaro M., Moreno-Loshuertos R., Fernandez-Silva P., Enriquez J.A., Laclaustra M. (2016). Adjusting MtDNA quantification in whole blood for peripheral blood platelet and leukocyte counts. PLoS ONE.

[27] Barret B., Tardieu M., Rustin P., Lacroix C., Chabrol B., Desguerre I., Dolfus C., Mayaux M.-J., Blanche S., French Perinatal Cohort Study (2003). Persistent mitochondrial dysfunction in HIV-1-exposed but uninfected infants: Clinical screening in a large prospective cohort. AIDS.

[28] Brogly S.B., Ylitalo N., Mofenson L.M., Oleske J., Van Dyke R., Crain M.J., Abzug M.J., Brady M., Jean-Philippe P., Hughes M.D. (2007). In utero nucleoside reverse transcriptase inhibitor exposure and signs of possible mitochondrial dysfunction in HIV-uninfected children. AIDS.

[29] Shen J., Liberty A., Shiau S., Strehlau R., Pierson S., Patel F., Wang L., Burke M., Violary A., Coovadia A. (2020). Mitochondrial Impairment in Well-Suppressed Children with Perinatal HIV-Infection on Antiretroviral Therapy. AIDS Res. Hum. Retrovir..

[30] Alimenti A., Burdge D.R., Ogilvie G.S., Money D.M., Forbes J.C. (2003). Lactic acidemia in human immunodeficiency virus-uninfected infants exposed to perinatal antiretroviral therapy. Pediatr. Infect. Dis. J..

[31] Noguera A., Fortuny C., Muñoz-Almagro C., Sanchez E., Vilaseca M.A., Artuch R., Pou J., Jimenez R. (2004). Hyperlactatemia in human immunodeficiency virus-uninfected infants who are exposed to antiretrovirals. Pediatrics.

[32] Ekouevi D.K., Touré R., Becquet R., Viho I., Sakarovitch C., Rouet F., Towne-Gold B., Fassinou P., Leroy V., Blance S. (2006). Serum lactate levels in infants exposed peripartum to antiretroviral agents to prevent mother-to-child transmission of HIV: Agence Nationale de Recherches Sur le SIDA et les Hépatites Virales 1209 study, Abidjan, Ivory Coast. Pediatrics.

[33] Fernández Ibieta M., Cano J.M.B., Amador J.T.R., González-Tomé M.I., Martín S.G., Gómez M.N., de José M.I., Beceiro J., Iglesias E., Prieto L. (2010). In-utero antiretroviral exposure and mitochondrial toxicity in a cohort of uninfected infants born to HIV-1-infected women. An. Pediatr..

