# Supplementary material for: Health Outcomes at School Age among Children Who Are HIV-Exposed but Uninfected with Detected Mitochondrial DNA Depletion at One Year
Source: J Clin Med. 2020 Nov 16;9(11):3680. doi: 10.3390/jcm9113680 (PMC7696966; doi:10.3390/jcm9113680)
Supplement: Supplementary file 1 [file jcm-09-03680-s001.zip › jcm-972848-supplementary.docx]

**Supplementary Materials**

**Result details: Quality control of DNA**

Seven children from Burkina Faso and seven from Uganda failed the quality control after checking for nuclear DNA degradation at each time point and were so excluded from the analyses. All the values showed no correlation with the platelet count. Spearman’s Rho coefficients for Burkina Faso were -0.012 (*p* = 0.44) at day-7 and 0.06 (*p* = 0.68) at year-6. Spearman’s Rho coefficients for Uganda were 0.02 (*p* = 0.89) at day-7 and 0.009 (*p* = 0.96) at year-6. Hurtado’s formula confirmed that platelet count did not influence MCN as the median difference between the raw value and the adjusted value did not vary from more than 3.0% with an interquartile range of [1.8;4.0] at day-7 and 6.4% [4.5;8.1] at year-6.

**Supplementary Table S1.** Characteristics of CHEU from the PROMISE PEP trial enrolled in the study and CHEU not enrolled.

| Characteristic | CHEU enrolled  (*n* = 86) | CHEU not enrolled  (*n* = 173) | *p* Value ^a^ |
| --- | --- | --- | --- |
| **Socio-demographics** |  |  |  |
| **Age** (in years); median [IQR] | 6.0 [5.0-6.0] | 6.0 [5.0-6.0] | 0.35 |
| **Site**; *n* (%) |  |  | <0.01 |
| Burkina Faso | 43 (50.0) | 52 (30.1) |  |
| Uganda | 43 (50.0) | 121 (69.9) |  |
| **Gender**; *n* (%) |  |  | 0.69 |
| Male | 44 (51.2) | 44 (48.6) |  |
| **Anthropometrics** |  |  |  |
| **Weight** (kg); mean ± SD | 19.9 ± 2.9 † | 19.0 ± 2.8 | 0.01 |
| **Height** (cm); mean ± SD | 115.7 ± 6.2 † | 113.3 ± 6.5 | <0.01 |
| **WAZ**; mean ± SD | -0.5 ± 0.9 † | -0.7 ± 0.9 | 0.06 |
| **HAZ**; mean ± SD | -0.3 ±1.0 † | -0.6 ± 1.1 | 0.03 |
| **BMIZ**; mean ± SD | -0.4 ±0.9 † | -0.5 ± 0.9 | 0.78 |
| **Hematology** |  |  |  |
| **Hemoglobin** **concentration** (g/dL); *n* (%) |  |  | 0.86 |
| Normal >10.4 | 80 (93.0) | 163 (94.2) |  |
| Anemia ≤10.4 | 6 (7.0) | - |  |
| Mild [10.4-9.5[ | 5 (5.8) | 7 (4.1) |  |
| Moderate [9.5-8.5[ | 1 (1.2) | 2 (1.2) |  |
| Severe [8.5-6.5[ |  | 1 (0.6) |  |
| **Platelet count** (10^3^/mm^3^); mean ± SD | 358.4 ± 110.2 | 353.0 ± 114.2 | 0.72 |
| **Platelet count** (10^3^/mm^3^); *n* (%) |  |  | 0.70 |
| Normal >125 | 86 (100.0) | 169 (97.7) |  |
| Thrombocytopenia ≥ 125 | - | 4 (2.3) |  |
| Mild [125-100[ | - | 1 (0.6) |  |
| Moderate [100-50[ | - | 3 (1.7) |  |
| **Leucocyte count** (10^3^/mm^3^); *n* (%) |  |  | NA |
| Normal >2.5 | 86 (100.0) | 173 (100.0) |  |
| **Neutrophil count** (10^3^/mm^3^); *n* (%) |  |  | 1.00 |
| Normal >1.0 | 84 (97.7) | 169 (97.7) |  |
| Neutropenia ≤1.0 | 2 (2.3) | 4 (2.3) |  |
| Mild [1.0-0.79[ | 2 (2.3) | 3 (1.7) |  |
| Very severe <0.4 | - | 1 (0.6) |  |
| **Biochemistry**; *n* (%) |  |  |  |
| **LDH concentration** (ULN) |  |  | 0.31 |
| Normal < ULN | 27 (31.4) | 41 (23.7) |  |
| Abnormal ≥ ULN | 59 (68.6) | 132 (76.3) |  |
| Mild [1-2[xULN | 59 (68.6) | 130 (75.1) |  |
| Moderate ≥2xULN | - | 2 (1.2) |  |
| **ALT** **concentration** (ULN) |  |  | 0.54 |
| Normal < 1.25xULN | 84 (97.7) | 170 (98.3) |  |
| Abnormal ≥ 1.25xULN | 2 (2.3) | 3 (1.7) |  |
| Mild [1.25-2.5[xULN | 1 (1.16) | 3 (1.7) |  |
| Moderate [2.5-5.0[xULN | 1 (1.16) | - |  |
| **Medical events**; *n* (%) |  |  |  |
| **Clinical consultation without admission during the last year** |  |  | 0.61 |
| Yes | 56 (65.9) † | 118 (69.0) ‡ |  |
| **Hospital admission since week-50** |  |  | 0.75 |
| Yes | 26 (31.3) § | 57 (33.3) ‡ |  |
| **Child ARV prophylaxis** |  |  | 0.27 |
| Lamivudine | 41 (47.7) | 95 (54.9) |  |
| Lopinavir/ritonavir | 45 (52.3) | 78 (45.1) |  |
| **Neuropsychological assessment** |  |  |  |
| **SDQ-25**; median [IQR] | 6.0 [3.0;9.0] † | 4.0 [2.0;8.0] † | 0.02 |
| **TOVA**; mean ± SD | 2.2 ± 0.8 £ | 2.2 ± 0.8 £ | 0.61 |
| **MABC-2**; mean ± SD | 78.2 ± 10.7 § | 79.2 ± 10.6 € | 0.75 |
| **KABC-II**; mean ± SD | 48.9 ± 12.9 § | 47.9 ± 12.5 ¤ | 0.59 |

^†^ one missing value, ^‡^ two missing values, ^§^ three missing values, ^£^ nine missing values, ^€^ four missing values, ^¤^ five missing values. ^a^ Chi-square test or Fisher’s exact test as appropriate and Wilcoxon signed-rank test or Student’s *t*-test. Abbreviations: CHEU, children who are HIV-exposed uninfected; IQR, interquartile range; SD, standard deviation; WAZ, weight-for-age z-score; HAZ, height-for-age z-score; BMIZ, body mass index z-score; LDH, lactate dehydrogenase; ALT, alanine aminotransferase; ULN, under limit of normal; ARV, antiretroviral; SDQ-25, strength and difficulties questionnaire; TOVA, test of variable of attention; MABC-2, movement assessment battery for children - second edition; KABC-II, Kaufman assessment battery for children - second edition; NA non-applicable.

**Supplementary Table S2.** Overall dynamic of mtDNA content.

|  |  |  | **Burkina Faso** | | **Uganda** | | **All** | |
| --- | --- | --- | --- | --- | --- | --- | --- | --- |
| **Follow-up time point** | **Group** | **PrEP** | ***n*** | **Median MCN [IQR]** | ***n*** | **Median MCN [IQR]** | ***n*** | **Median MCN [IQR]** |
| **day-7** | **Baseline MCN** | 3TC | 20 | 1014 [856;1415] | 21 | 1083 [950;1145] | 41 | 1083 [950;1145] |
|  |  | LPV/r | 23 | 1061 [938;1436] | 22 | 1084 [992;1198] | 45 | 1084 [992;1198] |
|  |  | **All** | **43** | **1053 [898;1436]** | **43** | **1083 [960;1180]** | **86** | **1082 [938;1253]** |
| **week-50** | **Increase of MCN from day-7** | 3TC | 6 | 1604 [1172;2093] | 10 | 1235 [1101;1792] | 16 | 1260 [1123;1876] |
|  |  | LPV/r | 8 | 1416 [1073;1684] | 11 | 1319 [1098;1763] | 19 | 1319 [1098;1729] |
|  |  | **All** | **14** | **1416 [1101;1776]** | **21** | **1261 [1101;1763]** | **35** | **1300 [1100;1776]** |
|  | **Decrease of MCN from day-7** | 3TC | 14 | 795 [657;918] | 11 | 470 [365;628] | 25 | 657 [436;855] |
|  |  | LPV/r | 15 | 793 [622;986] | 11 | 770 [6337;972] | 26 | 781 [637;972] |
|  |  | **All** | **29** | **793 [655;918]** | **22** | **632 [436;813]** | **51** | **742 [550;888]** |
| **year-6** | **Increase of MCN from week-50** | 3TC | 7 | 957 [642;1294] | 12 | 994 [822;1168] | 19 | 957 [808;1183] |
|  |  | LPV/r | 13 | 1279 [1091;1526] | 11 | 965 [869;1182] | 24 | 1099 [913;1459] |
|  |  | **All** | **20** | **1124 [872;1430]** | **23** | **965 [836;1182]** | **43** | **1027 [869;1385]** |
|  | **Decrease of MCN from week-50** | 3TC | 13 | 789 [617;904] | 9 | 1133 [976;1194] | 22 | 891 [623;1157] |
|  |  | LPV/r | 10 | 793 [665;945] | 11 | 900 [771;1280] | 21 | 867 [720;997] |
|  |  | **All** | **23** | **789 [647;945]** | **20** | **1014 [792;1237]** | **43** | **877 [689;1132]** |

Abbreviations: mtDNA, mitochondrial DNA; PrEP, pre-exposure prophylaxis; MCN, mitochondrial DNA copy number per cell; IQR, interquartile range; 3TC, lamivudine; LPV/r, lopinavir/ritonavir.

**Supplementary Table S3.** Characteristics at week-50 of the CHEU with depletion between the PROMISE PEP and the PROMISE M&S trials.

| Characteristic | CHEU with depletion M&S  (*n* = 17) | CHEU with depletion PEP  (*n* = 28) | *p* Value ^a^ |
| --- | --- | --- | --- |
| **Socio-demographics;** *n* (%) |  |  |  |
| **Site** |  |  | 0.67 |
| Burkina Faso | 9 (52.9) | 13 (46.4) |  |
| Uganda | 8 (47.1) | 15 (53.6) |  |
| **Gender** |  |  | 0.37 |
| Male | 8 (47.06) | 17 (60.7) |  |
| **Anthropometrics** |  |  |  |
| **Weight** (kg); mean ± SD | 8.1 ± 1.1 | 8.3 ± 1.1 | 0.52 |
| **Height** (cm); mean ± SD | 73.2 ± 3.9 | 72.9 ± 4.1 | 0.83 |
| **WAZ**; mean ± SD | -1.4 ± 1.3 | -1.2 ± 1.3 | 0.76 |
| **HAZ**; mean ± SD | -0.9 ± 1.2 | -1.1 ± 1.3 | 0.56 |
| **WHZ**; mean ± SD | -1.2 ± 1.3 | -0.91 ± 1.2 | 0.42 |
| **Underweight** (WAZ<2); *n* (%) | 6 (35.3) | 8 (28.6) | 0.74 |
| **Stunting** (HAZ<2); *n* (%) | 3 (17.7) | 7 (25.0) | 0.72 |
| **Wasting** (WHZ<2); *n* (%) | 4 (23.5) | 5 (17.9) | 0.71 |
| **Gestational age** (weeks); mean ± SD | 39.4 ± 2.0 | 38.7 ± 1.8 | 0.21 |
| **Preterm birth** (weeks); *n* (%) |  |  | 0.69 |
| No prematurity ≥ 37 | 15 (88.2) | 22 (78.6) |  |
| Prematurity < 37 | 2 (11.8) | 6 (21.4) |  |
| **Hematology ^b^** |  |  |  |
| **Hemoglobin concentration** (g/dL); mean ± SD  **Hemoglobin** **concentration** (g/dL); *n* (%) | 9.6 ± 1.2 † | 9.9 ± 1.1 ‡ | 0.34  0.75 |
| Normal >10.9 | 1 (6.7) † | 4 (16.0) ‡ |  |
| Anemia ≤10.9 | 14 (93.3) † | 21 (84.0) ‡ |  |
| Mild [10.0-10.9[ | 6 (40.0) † | 12 (48.0) ‡ |  |
| Moderate [9.0-10.0[ | 5 (33.3) † | 5 (20.0) ‡ |  |
| Severe [7.0-9.0[ | 3 (20.0) † | 4 (16.0) ‡ |  |
| **Platelet count** (10^3^/mm^3^); mean ± SD | 422.3 ± 203.1 † | 354.4 ± 169.4 ‡ | 0.26 |
| **Platelet count** (10^3^/mm^3^); *n* (%) |  |  | 1.00 |
| Normal >125 | 13 (86.7) † | 22 (88.0) ‡ |  |
| Thrombocytopenia ≥ 125 | 2 (13.3) † | 3 (12.0) ‡ |  |
| Mild [125-100[ | 1 (6.7) † | 1 (4.0) ‡ |  |
| Moderate [100-50[ | 1 (6.7) † | 2 (8.0) ‡ |  |
| **Leucocyte count** (10^3^/mm^3^); *n* (%) |  |  | NA |
| Normal >2.5 | 15 (100.0) † | 25 (100.0) ‡ |  |
| **Neutrophil count** (10^3^/mm^3^); *n* (%) |  |  | 1.00 |
| Normal >1.3 | 14 (93.3) † | 21 (84.0) ‡ |  |
| Neutropenia ≤1.3 | 1 (6.7) † | 4 (16.0) ‡ |  |
| Mild [1.0-1.3[ | 1 (6.7) † | 1 (4.0) ‡ |  |
| Moderate [0.75;1.0[ | - | 1 (4.0) ‡ |  |
| Severe [0.5;0.75[ | - | 1 (4.0) ‡ |  |
| Very severe <0.5 | - | 1 (4.0) ‡ |  |
| **Biochemistry ^b^;** *n* (%) |  |  |  |
| **ALT** **concentration** (ULN) |  |  | 0.62 |
| Normal < 1.25xULN | 12 (85.7) ‡ | 22 (91.7) § |  |
| Abnormal ≥ 1.25xULN | 2 (14.3) ‡ | 2 (8.3) § |  |
| Mild [1.25-2.5[xULN | 2 (14.3) ‡ | 2 (8.3) § |  |
| **Medical events;** *n* (%) |  |  |  |
| **Child ARV prophylaxis** |  |  | 0.62 |
| Lamivudine | 11 (64.7) | 16 (57.1) |  |
| Lopinavir/ritonavir | 6 (35.3) | 12 (42.9) |  |

^†^ two missing values, ^‡^ three missing values, ^§^ four missing values. ^a^ Chi-square test or Fisher’s exact test as appropriate and Student’s *t*-test. ^b^ Data was at week 38 postpartum because few data were available at week-50 for hematological and biochemical parameters. Abbreviations: CHEU, children who are HIV-exposed uninfected; IQR, interquartile range; SD, standard deviation; WAZ, weight-for-age z-score; HAZ, height-for-age z-score; WHZ, weight-for-height z-score; ALT, alanine aminotransferase; ULN, under limit of normal; ARV, antiretroviral; NA non applicable.
